# Endoscopic Measurement of Polyp Size Using a Novel Calibrated Hood

**DOI:** 10.1155/2014/714294

**Published:** 2014-06-30

**Authors:** Keiichiro Kume, Tatsuyuki Watanabe, Ichiro Yoshikawa, Masaru Harada

**Affiliations:** Third Department of Internal Medicine, School of Medicine, University of Occupational and Environmental Health, 1-1 Iseigaoka, Yahatanishi-ku, Kitakyushu 807-8555, Japan

## Abstract

*Background*. Although the size of colon polyps is an important risk factor for colorectal cancer, a standardized measurement technique has yet to be determined. In clinical practice, most endoscopists estimate polyp size by uncertain visual estimation; however, colonoscopic polypectomy is indicated for adenomatous polyps more than 5 mm in diameter. We have therefore developed a novel calibrated hood that enables accurate measurement of polyp size during colonoscopy. *Method*. We compared prepolypectomy estimates using the calibrated hood against measurements of preformalin-fixed samples immediately after polypectomy. *Results*. Sixty-five polyps removed from 44 patients were included in the present study. The mean size of polyps was significantly larger at prepolypectomy (6.06 ± 1.23 mm) than after polypectomy (5.48 ± 1.31 mm, *P* < 0.05). *Conclusion*. Accurately measuring the size of polyps during colonoscopy is important, since polyps are shrunk by polypectomy. Attaching the calibrated hood appears useful in the measurement of polyp size to determine indications for polypectomy in patients with colon polyps.

## 1. Introduction

Patients with larger colon polyps may be at greater risk for the development of colon cancer than those with small polyps with diameters of 5 mm or smaller [1–3]. Accurate assessment of polyp size is therefore important in assessing cancer risk. However, a standardized technique to measure polyp size in clinical practice has yet to be determined. The National Polyp Study used endoscopic estimation of polyp size with open biopsy forceps [3]. In another study, the pathologists' measurements were used [1], but the specific technique was not described [4]. In usual clinical practice, most endoscopists estimate polyp size simply by visual estimation. In Japan, colonoscopic polypectomy is indicated for adenomatous polyps over 5 mm in diameter [5]. Variations in the methodology applied to determine polyp size make it difficult to compare data between clinical studies for analysis and to decide the indications for polypectomy.

**Figure 1:**
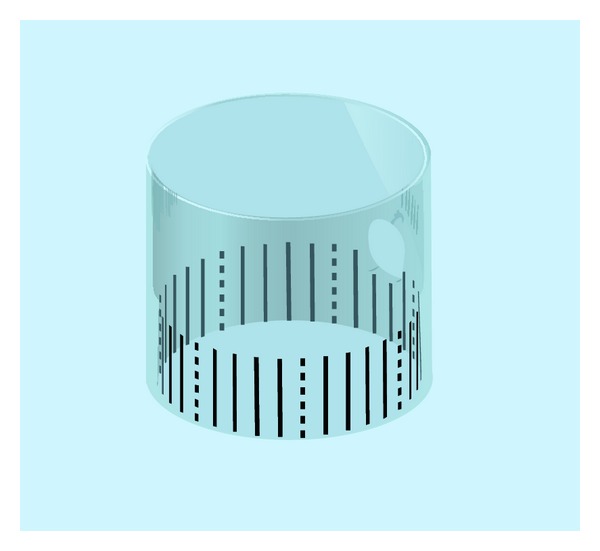
The calibrated hood was fabricated by attaching a graded plastic film to the inner circumference of the transparent hood. The calibrations consisted of unbroken lines marked on the plastic film at 1 mm intervals and broken lines at 5 mm intervals.

Cap-assisted colonoscopy is associated with improved detection of colorectal polyps and higher cecal incubation rate than standard colonoscopy [6–8]. We have therefore developed a calibrated hood for use in cap-assisted colonoscopy (design right and patent pending in Japan, but not commercial product). A calibrated plastic film is attached to the inner circumference of the transparent hood, which is then fitted to the tip of the endoscope, enabling simple measurement of colonic polyps (Figure 1).

We compared prepolypectomy estimates using the calibrated hood against measurements of preformalin-fixed samples immediately after polypectomy.

## 2. Methods

All study protocols were approved by the institutional review board at our university. Colonoscopy was performed for standard clinical indications after obtaining informed consent. Patients showing adenomatous polyps over 5 mm in diameter were enrolled in the present study. All adenomatous polyps more than 5 mm in diameter were removed. Polyps were excluded from the study if the polyp size in colonoscopy using the calibrated hood was revealed as less than 5 mm or more than 9 mm in diameter. Polyps that were fragmented were excluded. A video colonoscope (CF-Q260A, H260A, Q240A, or Q240; Olympus, Tokyo, Japan) was used.

The calibrated hood was fabricated by attaching a graded plastic film to the inner circumference of a distal attachment (D201; Olympus Co. Ltd., Tokyo, Japan). The calibrations consisted of unbroken lines marked on the plastic film at 1 mm intervals and broken lines at 5 mm intervals (Figure 1).

**Figure 2:**
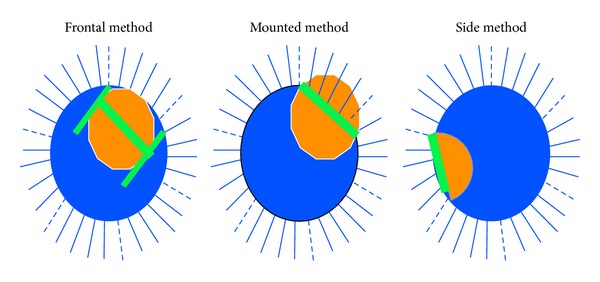
Three methods to measure polyp size using the calibrated hood. (1) “Frontal method”: the calibrated hood is placed directly over the top of the polyp, and the inner wall of the hood is brought into close contact with the side of the polyp. The diameter of the polyp is then calculated by envisioning horizontal lines extending from the hood's calibrations. (2) “Mounted method”: the rim of the calibrated hood is placed over the diameter of the polyp. (3) “Side method” (sessile polyps only): the inner wall of the hood is brought into close contact with the diameter of the polyp base.

The calibrated hood was used to measure polyp diameter according to one of the following three methods (Figure 2).

1. “Frontal method”: the calibrated hood is placed directly over the top of the polyp, and the inner wall of the hood is brought into close contact with the side of the polyp. The diameter of the polyp is then calculated by envisioning horizontal lines extending from the hood's calibrations.
2. “Mounted method”: the rim of the calibrated hood is placed on top of the polyp's diameter.
3. “Side method” (sessile polyps only): the inner wall of the hood is brought into close contact with the diameter of the polyp base.

Methods were selected based on whichever approach would allow the easiest measurement of polyp size.

The polyp was then excised using a standard snare electrocautery technique or endoscopic mucosal resection. The excised polyp was retrieved with a tri-pronged polyp grasper or suctioned through the endoscopic channel into a polyp trap. The retrieved polyp was measured immediately with a ruler.

**Figure 3:**
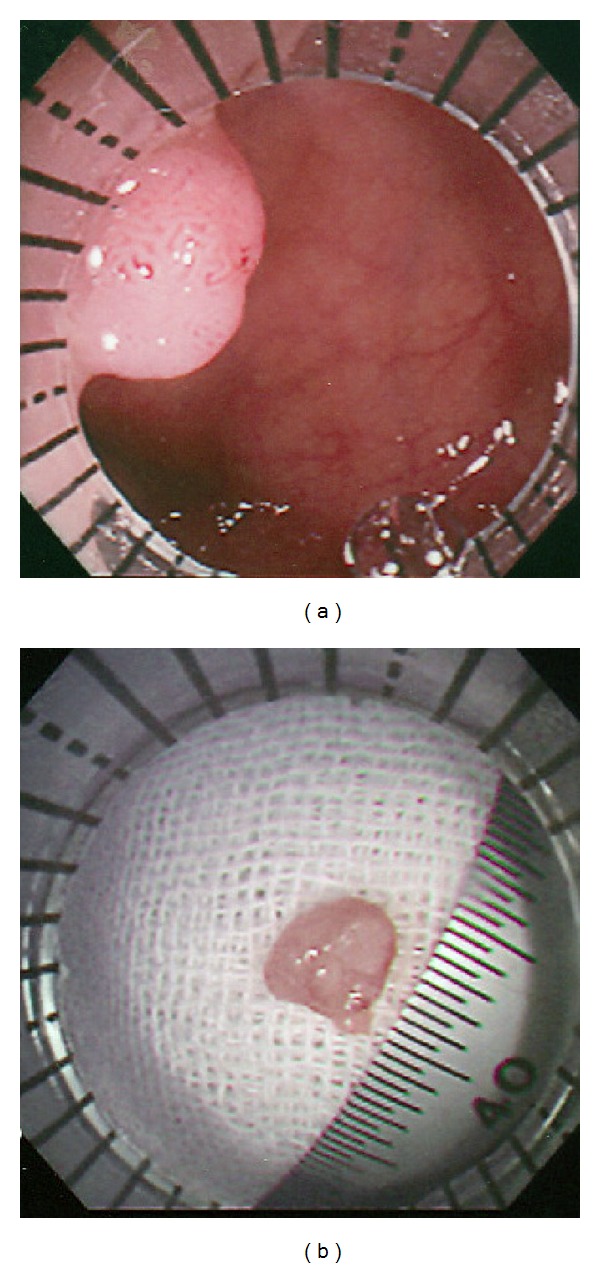
(a) A sessile polyp with a diameter of 6 mm was measured using the side method. (b) The retrieved polyp with a diameter of 5 mm was measured using a ruler.

An example with measurement of polyp size is shown in Figure 3. A sessile polyp changed from 6 mm in endoscopic measurement using the side method as in Figure 3(a) to 5 mm in postpolypectomy measurement as in Figure 3(b). 

### 2.1. Precision of the Calibrated Diameter

The calibrated diameter and actual polyp size were determined based on the relation between the arc and chord, as designated by the following formula:

(1) $$\begin{array}{c} y=\varphi \sin\left(\frac{x}{\varphi }\right), \end{array}$$

where *φ* is hood diameter, *y* is actual polyp diameter, and *x* is calibrated diameter.

**Figure 4:**
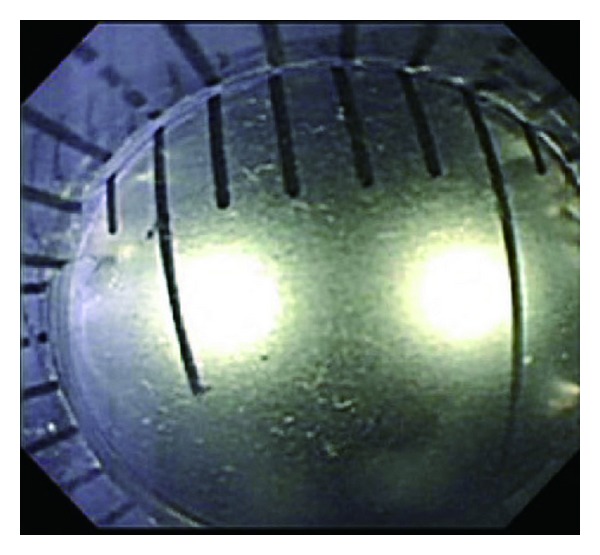
The calibrated hood in endoscopic view was placed directly over the ruler.

While there were slight differences due to hood diameter, the error between the calibrated measurement of 5 mm or 6 mm colonic polyps and actual polyp size was 0.1 or 0.2 mm, which was deemed clinically acceptable (Figure 4). At a calibrated measurement of 9 mm, this error became ≥0.5 mm, so 8 mm was set as the upper measurement limit for the hood.

### 2.2. Statistical Analysis

Results are expressed as mean ± standard deviation. Mann-Whitney *U* test with Bonferroni correction was used for statistical analysis. Values of *P* < 0.05 were considered to represent significant differences between the studied samples. Data were analyzed using StatView version 5 software.

## 3. Results

One hundred eleven adenomatous polyps were detected from 61 patients by eight endoscopists between July 2012 and May 2013. Ninety-two polyps more than 5 mm in diameter were removed. Nineteen polyps less than 5 mm, 19 polyps more than 9 mm in diameter, and eight polyps with fragmentation were excluded from this study. Overall, 65 polyps removed from 44 patients were included in the present study.

Mean size of polyps was 6.06 ± 1.23 mm in prepolypectomy estimates using the calibrated hood and 5.48 ± 1.31 mm in measurements of preformalin-fixed samples immediately after polypectomy measurement using a ruler (*P* = 0.0001).

## 4. Discussion

Decisions regarding indications of polypectomy for colon polyps are typically based on a prompt assessment of polyp size during colonoscopic inspection. Use of endoscopic devices capable of determining accurate polyp size is therefore essential during such inspections. However, the visual estimation of polyp size performed by most endoscopists in Japan is inevitably inaccurate, resulting in the resection of polyps that do not meet the indicated polypectomy criterion of ≥5 or 6 mm. Open biopsy forceps usually have a diameter of 8 mm when opened and can therefore be used to quickly assess polyps of this size [3, 9–11], but other sizes require visual estimation based on comparison with the forceps. The typical linear probe has markings at 2 mm intervals and is thus unsuitable for measuring small lesions and is rarely used for colonic polyps. Both the open biopsy forceps and linear probe methods require insertion into and removal from the forceps channel for each measurement; they also offer poor maneuverability. Moreover, the curved configuration of the sigmoid colon means that the colonoscope often deviates while attempting these methods, causing the colonoscopist to lose sight of the target polyp. We therefore sought to overcome these hurdles and combined the potential functions of an endoscopic hood by developing a calibrated hood that enabled accurate measurement of polyp size during colonoscopy.

The calibrated hood has a measurement limit of 8 mm, so it was used to measure the size of polyps in the measurable and resectable range of 5–8 mm prior to polypectomy and then compared with the preformalin-fixed measurement immediately after polypectomy. The results showed that measurements* in vivo* using the calibrated hood were significantly larger than those after polypectomy. Several studies have compared prepolypectomy estimates using the typical linear probe against measurements of preformalin-fixed samples immediately after polypectomy, and, in all cases, the prepolypectomy estimates were larger [9–11]. Those studies used the measurements of preformalin-fixed samples as reference values when referring to the accuracy of prepolypectomy estimates, so the findings are not useful for prepolypectomy assessments. However, our study using the calibrated hood is superior because it enabled accurate determination of polyp size prior to treatment while demonstrating similar results to previous studies.

In these investigations, the postpolypectomy, preformalin-fixed measurement was used as the basis for comparison. It has been suggested that vascular collapse after severing the polyp from its blood supply, polyp desiccation from cautery, or compression of the polyp during removal with a grasper or by suctioning through the endoscope may all contribute to reduced polyp size at postpolypectomy, preformalin-fixed measurement and may explain the endoscopists' overestimations of polyp size in clinical studies [9–13]. This is in contrast with* in vitro* studies, which suggest that endoscopists underestimate polyp size.

Although the calibrated hood has a measurement limit of 8 mm, in Japan, colonoscopic polypectomy is indicated for polyps ≥5 mm in diameter, while Atkin et al. proposed an alternative benchmark of ≥6 mm [1]. In other words, the capability to promptly assess whether a polyp is ≥5 (or ≥6) mm, which has heretofore primarily been performed visually, is the most important challenge in clinical colonoscopy. In conclusion, the use of a calibrated hood as proposed in the present study would provide a useful way to determine whether to perform polypectomy in patients with colon polyps.
